# Dissociation Between Instrumented Mobility and Self-Reported Function in Adults Aged 70 Years or Older After Unilateral Total Knee Arthroplasty: A Preliminary Longitudinal Study Using an Inertial Sensor and the WOMAC

**DOI:** 10.3390/jfmk11040420

**Published:** 2026-10-14

**Authors:** Andrei Machado Viegas da Trindade, Leonardo Pinheiro Rezende, Helder Rocha da Silva Araújo, Junichiro Sado, Karla Cristina Naves de Carvalho, Heytor Mendes Camargo Costa, Rafael Abrantes Jacinto, Allana Palma Fabricante, Veronica Cimolin, Rodolfo Borges Parreira, Claudia Santos Oliveira

**Affiliations:** 1 Faculty of Medicine, University Evangelica of Goiás, Anápolis 75083-515, GO, Brazil; andrei.trindade@einstein.br (A.M.V.d.T.); medkarcri@yahoo.com.br (K.C.N.d.C.); 2 Department of Orthopedics Surgery, Municipal Hospital of Aparecida de Goiânia (HMAP), Aparecida de Goiânia 74936-600, GO, Brazil; junichiro.sado@einstein.br (J.S.J.); heytormendesz@gmail.com (H.M.C.C.); rafaelabrantes18@hotmail.com (R.A.J.); allanapalma@hotmail.com (A.P.F.); 3 Health Sciences Program, Santa Casa de São Paulo School of Medical Sciences, São Paulo 01221-020, SP, Brazil; csneuro@gmail.com; 4 Graduate Program in Health Sciences, Federal University of Goiás (UFG), Goiânia 74605-050, GO, Brazil; drhelderrocha@hotmail.com; 5 Hospital Israelita Albert Einstein, São Paulo 05652-900, SP, Brazil; 6 Human Movement and Rehabilitation Post Graduation Program, Evangelical University of Goiás (UniEVANGELICA), Anápolis 75083-515, GO, Brazil; rodolfofcmscsp@gmail.com; 7 Department of Electronics, Information and Bioengineering, Politecnico di Milano, 20133 Milan, Italy; veronica.cimolin@polimi.it; 8 IRCCS Istituto Auxologico Italiano, San Giuseppe Hospital, Piancavallo, 28823 Verbania, Italy

**Keywords:** gait analysis, inertial measurement units, older adults, patient-reported outcome measures, total knee arthroplasty, Timed Up and Go, minimal detectable change, wearable sensors, WOMAC

## Abstract

**Background and Objectives:** Total knee arthroplasty (TKA) reliably reduces pain and improves self-reported function, yet objective mobility may remain impaired in older adults. We examined the relationship between inertial-sensor-based mobility and the Western Ontario and McMaster Universities Osteoarthritis Index (WOMAC) in adults aged 70 years or older after unilateral TKA. **Methods:** In this observational longitudinal study, 25 participants were assessed at up to three postoperative time points (M1, first available assessment; M2, 2024 follow-up; M3, 2025 follow-up). Outcomes were the instrumented Timed Up and Go (TUG) test with segmented turning variables, the 2-Minute Walk Test (2MWT), spatiotemporal gait variables recorded with a trunk-mounted inertial measurement unit, and the WOMAC. Paired changes in the instrumented outcomes were tested with Wilcoxon signed-rank tests under Benjamini–Hochberg false-discovery-rate (FDR) control and are reported with bootstrap confidence intervals (CIs); longitudinal change in the WOMAC was not tested, because the response format used at the first assessment could not be established from the available records, so WOMAC scores are used only within time point; associations used Spearman correlations with bootstrap CIs and leave-one-out influence diagnostics. Individual-level agreement was examined by standardising each of three externally validated objective outcomes against the inverted WOMAC total score within time point and classifying discordance across a range of cut-offs. Observed changes in the instrumented outcomes were benchmarked against published minimal detectable change (MDC) values. **Results:** Participants were 75.56 ± 4.00 years old and 64.0% were women; 14 were reassessed at M2 and 2 at M3. No instrumented global outcome changed after FDR adjustment, and the median changes in total TUG time (+0.07 s) and 2MWT distance (+12.80 m) remained below published MDC_95_ values (2.27 s and 14.96 m). Mid-turn duration shortened by a median of 0.80 s (95% CI −0.93 to −0.28) and mid-turn peak and mean angular velocities increased by 16.55°/s (8.00 to 30.10) and 7.40°/s (1.30 to 14.15), all with FDR-adjusted *p* = 0.036. At M2, the WOMAC total score was inversely associated with walking speed (rho = −0.762, 95% CI −0.973 to −0.307), cadence (rho = −0.761) and 2MWT distance (rho = −0.644); these three associations retained their sign and significance in every leave-one-out refit and changed little on adjustment for age, body mass index or postoperative duration, whereas all M1 associations were weak. Between 42.9% and 57.1% of participants fell outside a ±0.5 standardised concordance band at M2 depending on which validated outcome was used, with no consistent asymmetry in either direction, and the estimated prevalence ranged from 7.1% to 85.7% across plausible cut-offs. **Conclusions:** Group-level association between patient-reported and instrumented outcomes coexists with frequent individual-level discordance, but the magnitude of that discordance is not stably estimable in a cohort of this size. WOMAC scores should not be assumed to substitute for objective mobility testing after TKA, and confirmation in larger prospective cohorts is required.

## 1. Introduction

Total knee arthroplasty (TKA) is an effective treatment for advanced knee osteoarthritis when pain and functional limitation no longer respond to conservative management. In older adults, however, the outcome of surgery cannot be judged by pain relief alone: walking capacity, independence, dynamic stability, postural transitions and exercise tolerance are equally relevant to daily life. Contemporary work therefore supports a multidimensional postoperative assessment that combines patient-reported outcome measures (PROMs) with objective functional testing [1,2,3,4,5,6].

Self-reported instruments such as the Western Ontario and McMaster Universities Osteoarthritis Index (WOMAC) quantify pain, stiffness and perceived physical function, but they do not characterise gait biomechanics. Instrumented mobility studies, wearable-sensor reviews and direct comparisons of PROMs with laboratory or real-world function repeatedly show that subjective and objective recovery may move in the same direction at the group level while remaining only moderately associated within individuals [7,8,9,10,11,12,13,14,15,16,17,18,19]. A favourable WOMAC score cannot therefore be assumed to represent normalised mobility.

The WOMAC has established validity and interpretable thresholds in knee arthroplasty populations [20,21,22]. The Timed Up and Go (TUG) test and the 2-Minute Walk Test (2MWT) provide complementary information on basic mobility and submaximal walking capacity, and both have documented test–retest reliability and minimal detectable change values in patients with TKA [23,24,25,26]. Inertial measurement units (IMUs) add a further layer: by segmenting a composite task such as the TUG into its sit-to-stand, walking, turning and stand-to-sit phases, they quantify features of movement quality that a single stopwatch reading cannot resolve [27,28].

Restricting this study to adults aged 70 years or older has both a biological and a measurement rationale. Age-related sarcopenia, reduced postural control and slower central processing mean that the capacity to convert a pain-free joint into confident ambulation declines with age, so the same WOMAC score may correspond to poorer measured mobility in an older than in a younger recipient. Turning imposes coupled demands on balance, trunk control and limb loading that increase with age and are only partly reflected in the total duration of a mobility task. Older recipients are also the group in whom residual mobility limitation carries the greatest consequence for falls and independent living, yet arthroplasty cohorts reporting instrumented outcomes are typically younger and age-heterogeneous, so an age-specific estimate is not available from the existing literature. Restricting the cohort by age therefore sharpens the comparison between perceived and measured function, at the cost of limiting generalisability to younger recipients.

Evidence comparing fixed-bearing and rotating-platform implants has not demonstrated a consistent functional advantage for either design [29,30,31], whereas objective gait deficits are known to persist or evolve well beyond the first postoperative year [32,33,34,35]. The clinically useful question may therefore be less about which implant is superior and more about which patients show concordant, and which show discordant, objective and subjective recovery. The present study had three aims: (i) to describe the longitudinal course of instrumented mobility and WOMAC scores in adults aged 70 years or older after unilateral TKA; (ii) to quantify individual-level agreement between validated objective mobility outcomes and self-reported function, distinguishing association from agreement; and (iii) to interpret the observed changes against published measurement-error and clinical-relevance thresholds rather than against statistical significance alone. Loss to follow-up is reported explicitly and is kept distinct from missing outcome data among participants who attended an assessment.

## 2. Materials and Methods

### 2.1. Study Design and Reporting

This was a single-centre, observational, longitudinal, analytical study based on serial postoperative assessments recorded in an integrated clinical and sensor-based database of a specialised rehabilitation hospital. Reporting follows the Strengthening the Reporting of Observational Studies in Epidemiology (STROBE) recommendations [36]. Given the size of the cohort, the severity of attrition and the exploratory status of the segmented turning variables, the study is presented as preliminary and the analyses are framed accordingly.

### 2.2. Participants and Eligibility Criteria

Patients were eligible if they were aged 70 years or older, had undergone primary unilateral TKA, could be reliably matched across data sources, had a documented prosthesis category and had at least one functional or WOMAC assessment. Exclusion criteria were bilateral arthroplasty, revision arthroplasty, complete absence of both functional and self-reported outcomes, and inability to match the participant across databases.

Clinical descriptors beyond those reported in Section 3.1 were not available. Comorbidity burden, contralateral knee osteoarthritis severity, cognitive status, postoperative rehabilitation exposure, habitual physical activity, muscle strength, analgesic use and postoperative complications were not recorded during the original data collection and therefore could not be included either as descriptors or as covariates; they were not omitted from an available data set. Reasons for non-attendance at follow-up were likewise not recorded, which precludes any assessment of whether non-return was related to clinical deterioration. Surgical approach and operated side were available and are reported in Section 3.1.

### 2.3. Assessment Time Points and Follow-Up

Assessments are labelled as follow-up time points rather than as scenarios: M1 was the first available assessment for each participant, M2 the 2024 follow-up and M3 the 2025 follow-up; participant retention and the availability of each central outcome at each time point are given in Table 1. The source records contain no assessment-date field, so the interval between time points cannot be computed exactly. Reconstruction from date of surgery and recorded months since surgery places M1 within a window spanning August to October 2022 for 21 of the 25 participants, which together with the 2024 and 2025 collection years implies an M1–M2 interval of approximately 18 to 24 months and an M2–M3 interval of approximately 12 months. All intervals are therefore reported as approximatethe exact elapsed time for each participant is unknown, and no analysis is conditioned on it.; 

Time since surgery at M1 was heterogeneous (median 12 months, range 12–34 months). Participants entered observation at different postoperative stages, so M1–M2 change cannot be read as a common postoperative trajectory. To quantify rather than merely acknowledge this, months since surgery at M1 was carried through the analysis as a covariate (Section 2.8), and the principal associations are reported both unadjusted and adjusted for it (Section 3.7). Each participant contributed to a time point only if that assessment was actually performed; no values were carried forward. Framed precisely, the M1–M2 comparison describes change over a common calendar interval among participants who entered observation at different postoperative stages; it is not a shared recovery trajectory anchored to a common postoperative month.

### 2.4. Instrumentation and Testing Protocols

All functional tests were recorded with a single wireless IMU (G-Sensor/G-WALK; BTS Bioengineering, Garbagnate Milanese, Italy) fixed over the L5 spinous process with a semi-elastic belt. The unit measures 62 × 36 × 16 mm, weighs 60 g and integrates a triaxial accelerometer (±6 g), a triaxial gyroscope (±300°/s) and a triaxial magnetometer (±6 Gauss); it is factory-calibrated against gravitational acceleration. Signals were acquired at a sampling frequency of 50 Hz, transmitted by Bluetooth and processed with the manufacturer’s proprietary software (BTS G-Studio, version 2.6.12.0), which performs gait-event detection, task segmentation and computation of the reported variables. Placement was checked by the same examiner before every trial and the belt was tightened until no relative movement between the unit and the sacrum was observed during a preliminary walking bout. The concurrent validity and test–retest reliability of this system have been established against an instrumented walkway and against optoelectronic motion capture, with intraclass correlation coefficients between 0.85 and 0.99 for spatiotemporal parameters and excellent agreement for walking speed, cadence, stride length and stride duration [37,38].

Instrumented TUG. Participants rose from a standard chair without armrests, walked 3 m at a self-selected and safe speed, turned around a marker, walked back and sat down [23]. One familiarisation trial was followed by one recorded trial, and only the recorded trial was analysed. In addition to total duration, the software segments the task and returns the duration and the peak and mean angular velocity of the mid-turn (the 180° turn around the marker); these are the segmented variables analysed here. Turn detection is based on the trunk yaw angular-velocity signal: the turn interval is delimited by threshold crossings of the vertical-axis angular velocity, and peak and mean values are computed within that interval [27,28].

2MWT and walk test. The 2MWT was performed on a physically measured 10 m, level, unobstructed indoor walkway. Participants walked continuously for 2 min at a self-selected, safe pace, completing repeated 10 m traversals and making a 180° turn at each marked end of the course. The number of turns was not prespecified and varied according to the number of 10 m traversals completed within 120 s. Total distance was obtained from the sensor software. One familiarisation trial preceded one recorded 2MWT trial; no verbal or non-verbal encouragement was provided during the recorded trial beyond the initial standardised instructions, in order to preserve habitual walking behaviour. Spatiotemporal gait variables (walking speed and cadence) were obtained from a separate instrumented walk test performed at a self-selected speed along the same level walkway; this test likewise consisted of one familiarisation trial followed by one recorded trial. Across the instrumented TUG, 2MWT and walk test, participants wore their own non-standardised athletic shoes. Canes, crutches, walkers, other gait-assistive devices and orthoses were not permitted, and none were used.

The 2MWT was nominally a 2 min test. The recorded analysis duration at M1 had a median of 118.5 s (range 110–124 s), so the nominal 120 s divisor used to form average speed is an approximation rather than the realised test duration; the corresponding duration field was not recorded at M2 or M3. No participant took a rest pause at any visit.

A single recorded trial was analysed per test per visit. The original collection protocol captured one trial, so trial averaging is not possible retrospectively and no test–retest data exist for this cohort. Single-trial acquisition is more exposed to within-session and day-to-day variability than a trial-averaged protocol, particularly in older participants; it inflates measurement error, widens the uncertainty around every estimate reported here, and prevents estimation of laboratory-specific reliability, so published reliability values are used as external benchmarks (Section 2.7). This is a constraint of the source protocol rather than an analytical choice.

Published validation of this device covers spatiotemporal gait parameters and segment kinematics against instrumented-walkway and optoelectronic reference systems [37,38]. That validation does not extend to the segmented turning angular velocities analysed here, for which no device-specific validation is available. The turning variables are therefore treated as exploratory throughout, and the reliability of instrumented TUG subcomponents is known to be heterogeneous across studies [7,28].

### 2.5. Outcome Variables and Scoring

WOMAC. The WOMAC comprises 5 pain items, 2 stiffness items and 17 physical-function items, with domain scores formed by summation and a total score formed as their sum; higher scores indicate worse status. The item responses recorded at M1 span a wider range than those recorded at the 2024 and 2025 follow-ups: no item response above 2 occurs anywhere in the electronic follow-up forms across 1141 item responses, whereas at least one M1 respondent has domain scores that require responses above 2. We searched the available records to establish which response format was used at each administration. Item-level responses exist only for the 2024 and 2025 administrations, which used an identical electronic form; those exports record no response-option labels. For M1, only the computed domain scores survive, with no item-level data, no blank instrument and no data-collection protocol, so the response format used at M1 cannot be established from what is available.

Because the format at M1 is unknown, we do not treat M1 and later WOMAC scores as lying on a common metric. Numerically overlapping values would not settle the question: if the number of response categories or their verbal anchors differed between administrations, equal numbers need not carry equal meaning, and an argument about the range a format admits cannot establish commensurability. We therefore do not test or interpret longitudinal change in the WOMAC anywhere in this manuscript. WOMAC scores are reported as within-visit descriptives in the Results, and they enter the analysis only in within-time-point comparisons, where both measures come from the same administration and the question of format does not arise. The paired inferential analysis is confined to the instrumented outcomes, and no WOMAC change is benchmarked against minimal important differences. The nominal scale range is not asserted anywhere in this manuscript.

Domain and total scores were computed on a complete-case basis: a domain score was formed only when every item in that domain had been answered, and a total score only when all three domain scores were available. No item-level imputation was performed, and unanswered items were not treated as zero. At M3, this leaves the WOMAC total score unavailable for both returnees, because one answered 23 of the 24 items and the other only 2 (Section 3.3).

Walking speed. Three distinct speed variables are present in the source data and they are not interchangeable. First, 2MWT average speed is the walked distance divided by the nominal 120 s and describes sustained overground performance including turns. Second, 2MWT stride-derived mean speed is computed by the device from stride length and cadence during the same test. Third, walk-test speed is recorded during a separate short instrumented walk. Each is labelled explicitly wherever it appears in the text, tables and figures, and no value is transferred between them.

### 2.6. Individual-Level Agreement Between Objective and Self-Reported Function

Group-level correlation and individual-level agreement are different quantities: two measures can rank participants similarly while still disagreeing about where any individual participant sits. To examine agreement without constructing a new composite outcome, each of three externally validated objective outcomes was compared with the WOMAC separately: total TUG time, 2MWT distance and walk-test walking speed. Each has published measurement-error or clinical-relevance benchmarks (Section 2.7), and none requires a weighting decision (see Appendix A). Agreement is assessed from the distribution of individual differences between two standardised measures, following the difference-based logic of Bland and Altman [39], but without converting the comparison into limits of agreement in clinical units, which standardised scores cannot support.

z_ij_ = (x_ij_ − mean_j_)/SD_j_, d_i_ = z(objective)_i_ − z(−WOMAC total)_i_ (1)

Within each time point j, the objective outcome and the inverted WOMAC total score were converted to standardised scores so that higher values indicate better status on both, and the difference d_i was formed for every participant i with both measures available. A participant was classified as concordant when the absolute difference did not exceed a cut-off, as objective better than self-report when the difference exceeded it, and as self-report better than objective when the difference fell below its negative. Because no cut-off has been validated for this comparison, a value of 0.5 standardised units is reported alongside a sensitivity analysis spanning cut-offs from 0.1 to 1.5, and the stability of the resulting prevalence estimate is presented as part of the result rather than as a robustness footnote. Standardisation is performed within time point, so the difference describes relative position within the cohort assessed at that visit; its magnitude is not comparable across visits, the classification is therefore reported separately by time point, and no longitudinal change in the difference is modelled.

### 2.7. Benchmarks for Measurement Error and Clinical Relevance

No test–retest measurements were collected in the present cohort, so observed changes were interpreted against published benchmarks obtained in comparable populations. For total TUG time and 2MWT distance we used the minimal detectable change at the 95% confidence level (MDC95) reported in patients with TKA: 2.27 s for the TUG and 14.96 m for the 2MWT, derived from intraclass correlation coefficients of 0.98 and 0.97 and standard errors of measurement of 0.82 s and 5.40 m [26]. For walking speed, we used the established anchors for meaningful change in older adults: 0.05 m/s for a small and 0.10 m/s for a substantial change [40]. No benchmark is applied to the WOMAC. Published minimal important differences for the instrument are expressed on a 0–100 metric and their translation into raw points presupposes a known number of response categories, which is not available for M1 (Section 2.5); benchmarking WOMAC change would therefore assume the common metric we are unable to establish. For the segmented TUG variables and for cadence, for which no anchor-based MDC exists in this population, the distribution-based half-SD criterion was applied using the M1 standard deviation of the cohort [41]. Published reliability estimates for instrumented TUG subcomponents are heterogeneous and are systematically lower for turning and postural-transition phases than for total duration [7,28]; this asymmetry was taken into account when interpreting the segmented results.

### 2.8. Statistical Analysis

Continuous variables are summarised as mean ± standard deviation (SD) and median [interquartile range (IQR)]; categorical variables as n (%). Paired M1–M2 comparisons used the Wilcoxon signed-rank test, the median change and the paired standardised effect size (Cohen’s dz). Associations between sensor-derived outcomes and the WOMAC total score used Spearman’s rank correlation. Fixed-bearing and rotating-platform implants were compared with the Mann–Whitney U test and Cliff’s delta. Comparisons involving M3 are descriptive, because only two participants returned. Two-sided p values are reported, and the Benjamini–Hochberg procedure controlled the false discovery rate (FDR) within each family of related tests [42].

Paired M1–M2 changes were tested with the Wilcoxon signed-rank test, and median changes and paired standardised effect sizes are reported with 2.5th to 97.5th percentile bootstrap confidence intervals from 10,000 resamples. Spearman correlations are reported with both bootstrap and Fisher z-transformed confidence intervals, and with leave-one-out influence diagnostics in which the coefficient is recomputed with each observation removed in turn and the resulting range, sign stability and significance stability are reported.

Because the number of participants is small relative to the number of plausible confounders, a fully adjusted multivariable model is not estimable: with 14 paired observations, a regression carrying age, sex, body mass index, postoperative duration and an implant term would have fewer than three observations per parameter and would not yield interpretable coefficients. Covariate influence was therefore assessed one covariate at a time using partial Spearman correlations, which remove the rank association of a single covariate, and by stratification for sex. This quantifies whether a candidate confounder accounts for an observed association without asserting an adjusted effect that the data cannot support. Attrition was examined by comparing every available baseline characteristic between participants reassessed at M2 and those not reassessed, and by repeating the principal paired analyses with inverse-probability-of-attrition weights estimated from a logistic model for M2 return. The same procedure is not estimable at M3, where only two participants were reassessed.

### 2.9. Sample Size and Precision

The analysable sample was fixed by the size of the source cohort. Using the reference values adopted in our previous work in this population—an expected between-assessment difference in walking speed of 0.27 m/s with a standard deviation of the paired difference of 0.30 m/s, that is dz = 0.90, derived from instrumented gait measurements after primary TKA [7,32]—a two-sided paired comparison with α = 0.05 and 80% power requires 12 complete pairs; 14 complete pairs were available for the M1–M2 analysis. Because power calculations performed after the fact on observed effects are uninformative, we report instead the precision that was actually achieved: with 14 pairs, the study had 80% power to detect a standardised paired effect of |dz| ≥ 0.81, and it had 80% power to detect a correlation of |ρ| ≥ 0.57 with 22 cases and |ρ| ≥ 0.69 with 14 cases. Effects smaller than these could not be detected reliably, and the non-significant global results must be read in that light.

These calculations describe the effect sizes the available sample could detect; they are not a claim that the study is adequately powered. Only large paired effects and large correlations are detectable at these sample sizes, and moderate effects of clinical interest are not. Accordingly, no non-significant result in this manuscript is interpreted as evidence of absence, and every principal estimate is accompanied by a confidence interval so that the full range of values compatible with the data is visible.

### 2.10. Data Preparation

Data files were linked using normalised names and, where available, registration numbers. An additional 2025 file was incorporated to recover instrumented TUG values for participants reassessed at M3. Numeric values stored as text containing the “±” symbol were parsed while preserving the reported central value. No outcome imputation was performed, because absence at M3 represents loss to follow-up rather than an unrecorded measurement during an attended visit. Analyses were carried out on de-identified records.

Four source-data issues were identified during preparation and are resolved as follows. First, one participant’s M2 total TUG duration was stored as a text string combining the duration with the functional-mobility category; the numeric value was extracted and retained, which restores the M2 denominator to 14. Second, two records of the M2 instrumented TUG exist, one in the master collection workbook and one in the 2024 collection workbook, and they disagree for the four participants present in both; the 2024 workbook was treated as authoritative because it is the complete record for that campaign, whereas the master-workbook block covers only 5 of the 25 participants. Third, a short prosthesis-type code disagrees with the spelled-out implant field for two participants in the analytic cohort; the spelled-out field was retained because it is consistent with the recorded surgical approach in both cases. Fourth, the 2MWT analysis-duration column in the 2024 and 2025 workbooks duplicates the M1 column and was therefore treated as unavailable at those visits.

## 3. Results

### 3.1. Participant Characteristics

**Figure 1:**
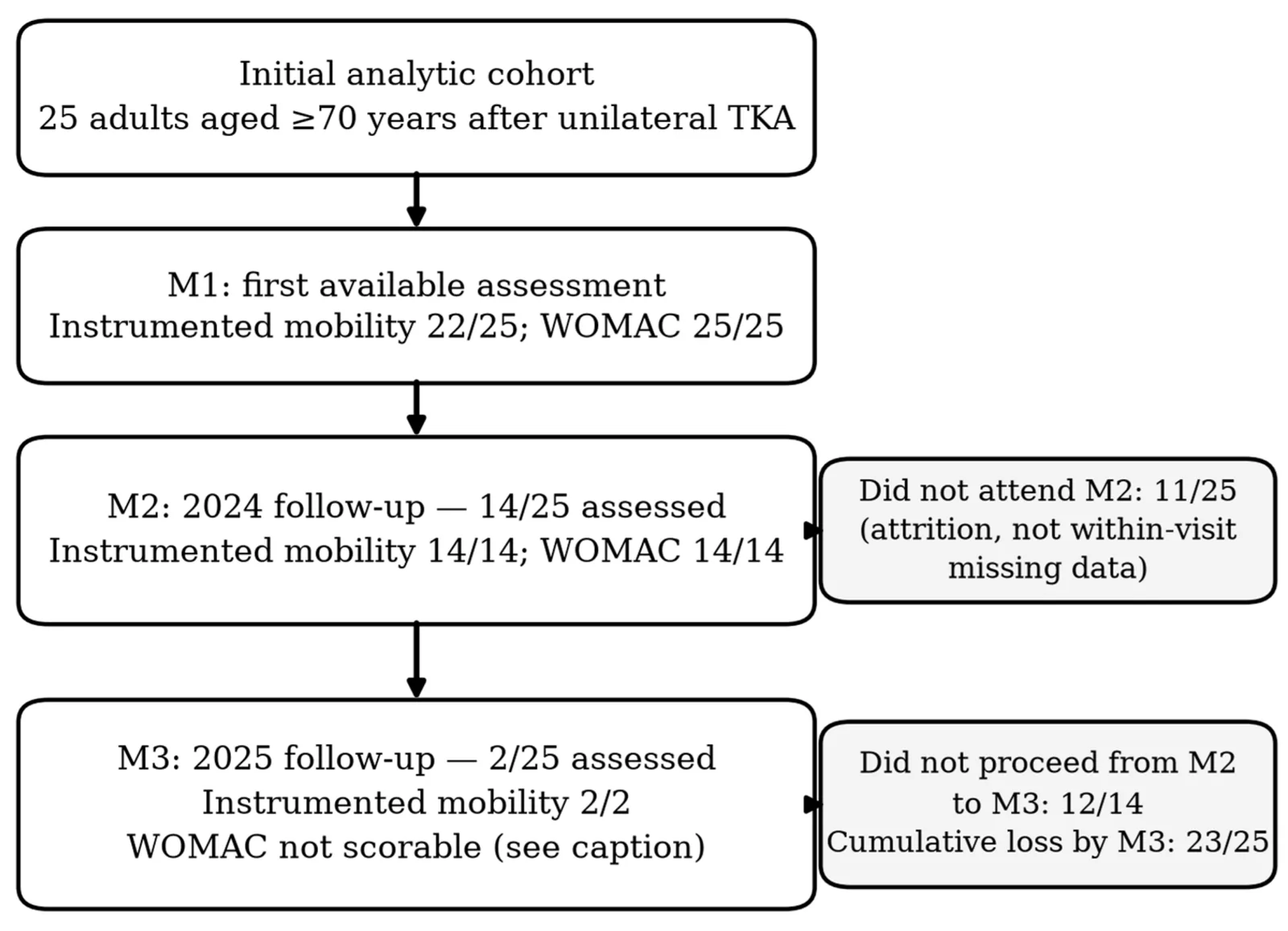
Participant flow through the three assessment time points. Denominators refer to the initial analytic cohort of 25. White boxes are assessment time points, shaded boxes are participants lost to follow-up, and arrowheads indicate progression through the study. Counts on the right are shown separately as the M2 non-attendance count, the M2-to-M3 transition count and the cumulative loss by M3. WOMAC scores could not be computed at M3 under complete-case scoring because one returnee answered 23 of the 24 items and the other only 2.

The analytic cohort comprised 25 adults aged 70 years or older. Mean age was 75.56 ± 4.00 years; 16 participants (64.0%) were women and mean body mass index (BMI) was 29.80 ± 5.09 kg/m^2^. Fifteen participants (60.0%) received a fixed-bearing implant and ten (40.0%) a rotating-platform implant (Table 2, Table 3 and Table 4). Participant flow is shown in Figure 1.

Implant type and surgical approach are completely confounded in this cohort: all 15 fixed-bearing procedures were performed through a transquadriceps approach and all 10 rotating-platform procedures through a subvastus approach (Fisher’s exact *p* < 0.001). The same pattern holds in the wider surgical register from which the cohort was drawn, where 56 of 57 procedures follow it. No analysis in this data set can therefore separate bearing design from surgical exposure, and any difference between implant groups must be read as a difference between two surgical packages rather than between two bearing designs.

### 3.2. Follow-Up Attrition

Retention was 14/25 (56%) at M2 and 2/25 (8%) at M3, corresponding to cumulative loss to follow-up of 23/25 (92%) by M3. Of the 14 participants reassessed at M2, 12 did not proceed to M3. Both M3 returnees had attended M2, so no participant skipped a visit and returned, and the cumulative and transition counts coincide.

Participants reassessed at M2 were compared with those not reassessed across all 16 available baseline characteristics (Table 5). No characteristic differed at conventional significance: the smallest unadjusted *p* value was 0.116 and the smallest FDR-adjusted *p* value was 0.736. This does not establish comparability. With 14 and 11 participants per group, the comparison can detect only large differences, so the correct reading is that differential attrition cannot be excluded, not that it is absent.

Repeating the principal paired analyses with inverse-probability-of-attrition weights left the conclusions unchanged. The weighted median M1–M2 changes were +0.02 s for total TUG time (unweighted +0.07 s), +13.40 m for 2MWT distance (+12.80 m), −0.06 m/s for walking speed (−0.04 m/s) and +17.20°/s for mid-turn peak angular velocity (+16.55°/s). The weighting model carried age, body mass index and months since surgery, giving 4.7 events per variable, and is itself exploratory; none of its covariates predicted return (all *p* > 0.27).

No attrition analysis is possible for M3. With two participants retained, baseline comparison and inverse-probability weighting are both uninformative, and the appropriate conclusion is that attrition bias at M3 cannot be evaluated at all rather than that retained and lost participants were comparable.

### 3.3. Longitudinal Description of Outcomes

Available-case descriptive values are presented in Table 6. The effective sample fell from 22 to 25 at M1 to 14 at M2 and 2 at M3, so the three columns describe progressively smaller and progressively more selected groups and should not be read as a trajectory. At M3, instrumented outcomes were available for both returnees, but the WOMAC total score could not be scored for either: one returnee answered 23 of the 24 items, leaving the physical-function domain incomplete, and the other answered only 2. WOMAC values are therefore reported at M3 only for the domains in which every item was answered.

### 3.4. Paired M1–M2 Comparison

None of the instrumented global outcomes—total TUG time, 2MWT distance, either 2MWT-derived speed, walk-test walking speed or cadence—changed detectably between M1 and M2 after FDR adjustment (Table 7), and every corresponding confidence interval is wide enough to include both clinically trivial and clinically relevant values. Three segmented turning variables did change: mid-turn duration shortened by a median of 0.80 s (95% CI −0.93 to −0.28), mid-turn peak angular velocity increased by 16.55°/s (8.00 to 30.10) and mid-turn mean angular velocity by 7.40°/s (1.30 to 14.15), each with an FDR-adjusted *p* value of 0.036 and a paired effect-size interval excluding zero. The FDR family comprised the eight instrumented outcomes listed in Table 7. Longitudinal change in the WOMAC is not tested, for the reason given in Section 2.5: the response format used at M1 cannot be established, so M1 and later scores may not share a common metric. WOMAC values by visit are given in Table 6 and are not to be read as change.

The direction of the global TUG result deserves comment, because total TUG time was numerically longer at M2 while the turning phase became shorter and faster. A change in how a task is executed need not appear in its total duration, since time saved in one phase can be spent in another. This observation is descriptive: the total-duration change was well within measurement error, and with 14 paired observations the turning result rests on a single trial per visit in a variable set for which no laboratory-specific reliability estimate exists.

### 3.5. Measurement Error and Clinical Relevance of the Observed Changes

Table 8 places each M1–M2 change alongside the corresponding measurement-error or clinical-relevance benchmark. Only the two mid-turn angular-velocity variables exceeded their threshold. Every other change—total TUG time, 2MWT distance, walking speed, cadence and all WOMAC domains—fell below the relevant MDC or minimal important difference, which is coherent with the absence of FDR-adjusted significance and indicates that these changes should not be interpreted as clinically meaningful, in either direction.

The margins by which the turning changes exceed their distribution-based thresholds are uneven. Mid-turn mean angular velocity exceeds its threshold by 0.03°/s, less than 0.5% of the threshold itself, and its confidence interval for the median change extends from 1.30 to 14.15°/s, so whether the true change exceeds the threshold is not resolved by these data. Mid-turn peak angular velocity exceeds its threshold by a wider margin, and mid-turn duration falls below its own. Distribution-based thresholds reflect the dispersion of this cohort rather than any patient-valued quantity, and no anchor-based estimate exists for segmented turning variables, so they are used here only as a scale for interpreting magnitude and not as evidence of clinical benefit.

### 3.6. M3 Follow-Up

Comparisons involving M3 are descriptive and are reported in full in the Supplementary Materials (Table S1 and Figure S1). Both returnees had longer total TUG times at M3 than at M2 (17.08 s and 27.00 s against 12.83 s and 17.92 s) and shorter 2MWT distances. The WOMAC could not be scored at M3 for either returnee, so no comparison between objective and self-reported function is possible at that visit. With two participants, no inference is available, and the M3 data are presented only to document what was collected.

### 3.7. Association Between Objective Mobility and the WOMAC

**Figure 2:**
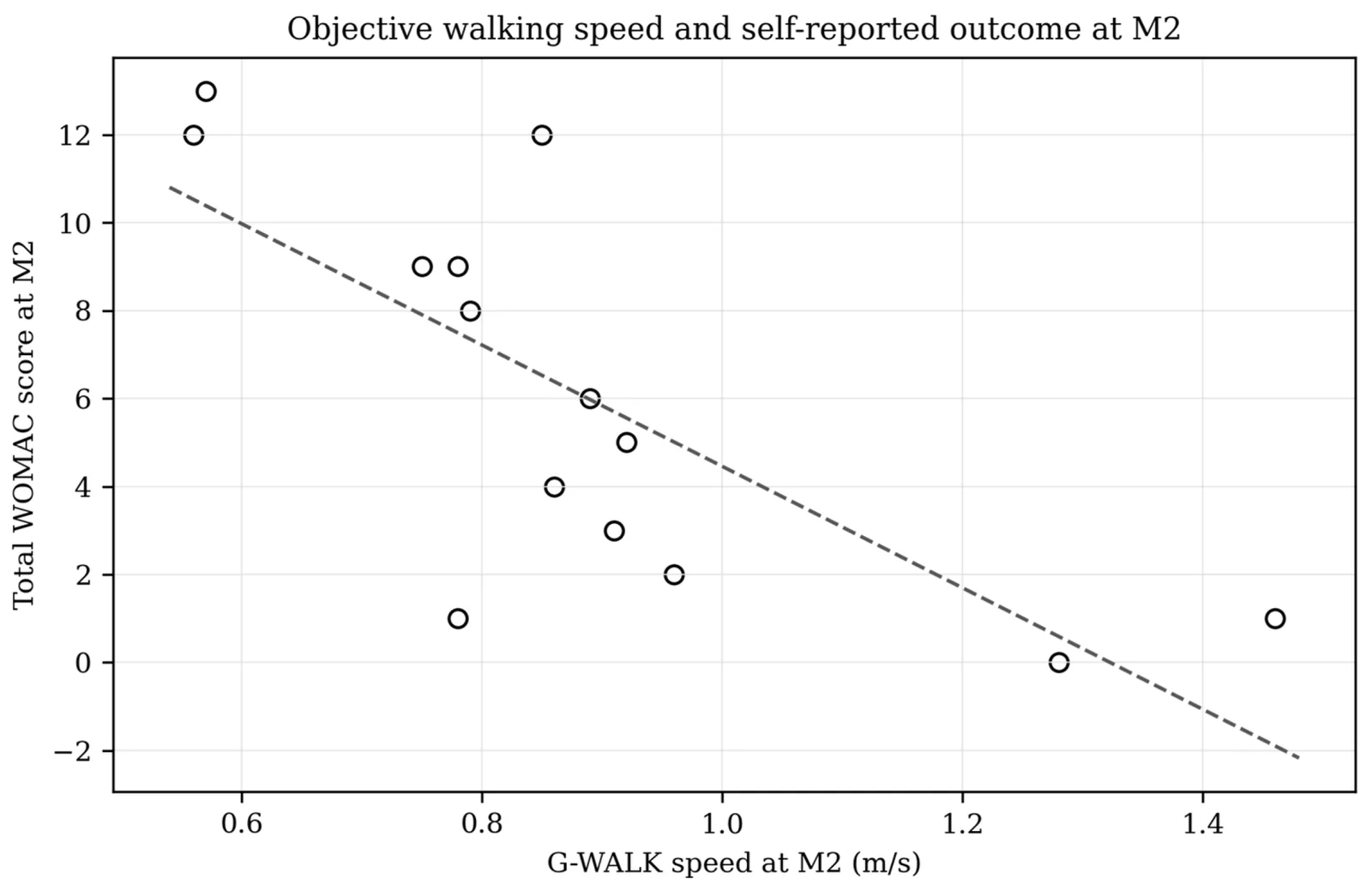
Association between walk-test walking speed and total WOMAC score at M2 (*n* = 14). The dashed line is a least-squares fit shown to indicate direction only; the reported association is the Spearman coefficient (rho = −0.762, 95% bootstrap CI −0.973 to −0.307). Each open circle is one participant. Lower WOMAC scores indicate better self-reported status.

At M1, correlations between objective mobility and the WOMAC total score were weak and none reached statistical significance; every confidence interval spans zero and includes both moderate positive and moderate negative values (Table 9). At M2, the WOMAC total score was inversely associated with walk-test walking speed (rho = −0.762, 95% bootstrap CI −0.973 to −0.307; FDR-adjusted p = 0.004), with cadence (rho = −0.761, −0.949 to −0.345) and with 2MWT distance (rho = −0.644, −0.901 to −0.124); the association with walking speed is shown in Figure 2. Fisher z-transformed confidence intervals for every coefficient are given in Table S3.

Leave-one-out diagnostics support these three associations. Removing any single participant leaves the coefficient between −0.915 and −0.710 for walking speed, between −0.860 and −0.701 for cadence and between −0.747 and −0.559 for 2MWT distance, with the sign preserved and significance retained in all 14 refits. The association with total TUG time (rho = +0.569) is not robust on the same test: its bootstrap interval includes zero (−0.013 to 0.896) and it loses significance in some leave-one-out refits, so it is not interpreted further. Partialling age, body mass index or months since surgery one at a time changed the M2 walking-speed association little (partial rho −0.774 to −0.807), and the association was present in both sexes on stratification (women rho = −0.550, *n* = 9; men rho = −0.564, *n* = 5), although each stratum is too small to interpret on its own. Removing the stiffness domain from the WOMAC total leaves the M2 walking-speed correlation unchanged at −0.762, because stiffness was zero for every paired participant and therefore contributes no variance; the domain is retained for comparability with published WOMAC totals but carries no discriminatory information in this cohort.

Repeating the correlation analysis against the WOMAC physical-function subscale rather than the total score gave the same picture, so the association does not depend on which self-report anchor is used. At M2 the subscale was associated with walking speed (rho = −0.782, 95% bootstrap CI −0.961 to −0.342; FDR-adjusted p = 0.004) and cadence (rho = −0.724, −0.914 to −0.307), both significant in all 14 leave-one-out refits; the association with 2MWT distance (rho = −0.600) was weaker than against the total score and not significant in every refit, and the association with total TUG time (rho = +0.532) was again not robust. At M1 all four associations were weak and none reached significance. The full subscale analysis is given in Supplementary Table S4.

### 3.8. Individual-Level Agreement Using Validated Outcomes

**Figure 3:**
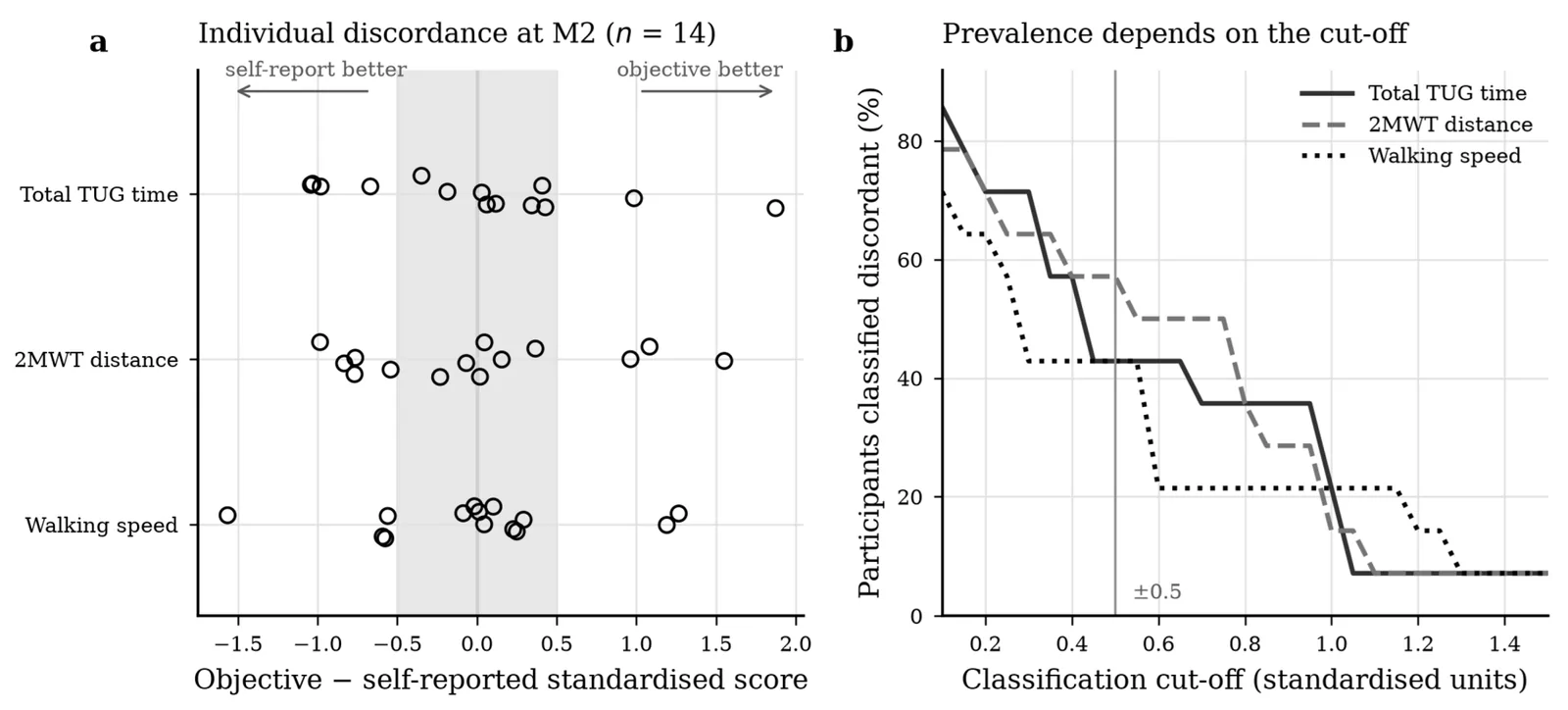
Individual-level agreement between validated objective outcomes and the WOMAC at M2 (*n* = 14). (**a**) Difference between the standardised objective outcome and the standardised inverted WOMAC total score for each participant, for each of the three validated outcomes; the shaded band is the ±0.5 standardised concordance band and each open circle is one participant and points are jittered vertically to separate ties. (**b**) Proportion of participants classified discordant as a function of the classification cut-off. No cut-off is externally validated for this comparison, and the prevalence estimate is not stable across the plausible range.

Individual-level agreement was examined separately for each of the three validated objective outcomes (Table 10, Figure 3). At M2, the proportion of participants falling outside a ±0.5 standardised concordance band was 42.9% using total TUG time, 57.1% using 2MWT distance and 42.9% using walking speed; at M1 the corresponding proportions were 63.6%, 63.6% and 72.7%. Frequent individual discordance is therefore present whichever validated outcome is used and is not an artefact of any single measure.

The direction of discordance was not consistently asymmetric. At M1, the two discordant categories were exactly balanced for all three outcomes (7 against 7 for total TUG time, 7 against 7 for 2MWT distance and 8 against 8 for walking speed). At M2, the self-report-better category was somewhat larger than the objective-better category (4 against 2 for total TUG time and for walking speed, 5 against 3 for 2MWT distance), but the counts are single digits and provide no reliable evidence of asymmetry in either direction.

The estimated prevalence depends strongly on the classification cut-off (Figure 3b). Across cut-offs from 0.1 to 1.5 standardised units, the proportion classified discordant at M2 ranges from 7.1% to 85.7% for total TUG time, from 7.1% to 78.6% for 2MWT distance and from 7.1% to 71.4% for walking speed. At the two additional cut-offs of ±0.3 and ±1.0 standardised units the M2 proportions were 71.4% and 21.4% for total TUG time, 64.3% and 14.3% for 2MWT distance, and 42.9% and 21.4% for walking speed; the corresponding M1 proportions were 77.3% and 31.8%, 77.3% and 45.5%, and 81.8% and 45.5%. Anchoring the same classification on the WOMAC physical-function subscale instead of the total score gave 57.1%, 57.1% and 42.9% outside the ±0.5 band at M2 and 72.7%, 63.6% and 77.3% at M1, so the qualitative finding is not specific to the total score (Supplementary Table S4). Because no cut-off is externally validated for this comparison, the qualitative finding that individual discordance is common is supported by these data, whereas any specific prevalence figure is not. Agreement is also expressed in standardised rather than clinical units, so the difference describes relative position within the cohort assessed at that visit and does not translate into seconds, metres or WOMAC points.

### 3.9. Comparison by Prosthesis Type

Because implant type is completely confounded with surgical approach in this cohort (Section 3.1) and each subgroup contains only 7 participants at M2, no comparison between bearing designs is interpretable. The exploratory comparison is reported in the Supplementary Materials (Table S2) for completeness. After FDR adjustment, no difference was detected between the groups on any outcome at either time point, and that absence must not be read as evidence of equivalence: the study was not designed as an implant comparison, prosthesis type was not a prespecified objective, and the confidence intervals are compatible with clinically important differences in either direction.

## 4. Discussion

The principal finding is that instrumented mobility and WOMAC-based self-reported function carried related but not interchangeable information in this cohort. At the 2024 follow-up, the WOMAC total score was strongly and inversely associated with walking speed, cadence and 2MWT distance, and these associations survived leave-one-out removal and single-covariate adjustment. At first assessment, the same associations were weak and indistinguishable from zero. Alongside this group-level association, between 42.9% and 57.1% of participants at M2 fell outside a standardised concordance band, and the proportion was similar or larger at M1. Association and agreement therefore behaved differently in the same data, which is the substantive observation this study supports.

The contrast between a numerically longer total TUG time and shorter, faster turns illustrates why segmented analysis can add information that a summary duration does not carry. Among the instrumented outcomes, only the turning variables changed detectably between M1 and M2, and their confidence intervals excluded zero. That said, these are the outcomes with the weakest external support in this study: the device’s published validation covers spatiotemporal gait parameters rather than segmented turning angular velocity, reliability of instrumented TUG subcomponents is heterogeneous across studies, a single trial was recorded per visit, and one of the three changes exceeded its distribution-based threshold by a negligible margin. An isolated signal in the most exploratory variable family, in 14 paired observations, is a hypothesis rather than a demonstrated physiological adaptation.

The prevalence of individual discordance could not be estimated stably. Varying the classification cut-off across its plausible range moved the M2 estimate between 7.1% and 85.7%, and no cut-off for this comparison has been externally validated. The qualitative conclusion, that a substantial fraction of older TKA recipients sit far from the cohort relationship between measured mobility and reported function, is robust to the choice of validated outcome; the numerical prevalence is not. Earlier reports of discordance between patient-reported and performance-based outcomes after arthroplasty have generally described its existence rather than quantified its frequency [16,17,19], and the present data suggest that quantification requires both a validated threshold and a substantially larger sample.

Two features of the design limit what can be inferred about mechanism. Implant type is completely confounded with surgical approach, so this cohort cannot speak to bearing design at all, and the subgroup comparison is reported only as Supplementary description. Postoperative timing at first assessment ranged from 12 to 34 months, so participants entered observation at different stages of recovery; adjusting for months since surgery did not materially change the principal associations, but adjustment cannot substitute for a common assessment schedule.

Clinically, these observations support measuring objective mobility alongside patient-reported outcomes in older TKA recipients rather than treating either as a proxy for the other. They do not support the identification of dissociation phenotypes, the use of any specific threshold to flag individual patients, or a change in follow-up practice. A prospective cohort with a common assessment schedule, repeated trials, recorded comorbidity and rehabilitation exposure, and a prespecified agreement threshold would be required before any of that became actionable.

Limitations. The initial cohort was small and attrition was severe, reaching 92% by M3, so the three-time-point design contributes little beyond the M1–M2 comparison and M3 is reported descriptively. Selection operated before enrolment as well as during follow-up: this is a single-centre cohort of older adults who were able to complete the test battery without assistive devices, and participants who required a cane, crutch, walker or orthosis were not permitted to use one during testing. The sample therefore under-represents the more mobility-impaired part of the population aged 70 years or older after TKA, which is precisely the group in whom discordance between reported and measured function might be largest. Postoperative timing was heterogeneous and exact assessment dates were unavailable. A single trial was recorded per test per visit, and no test–retest data exist, so laboratory-specific reliability could not be estimated. The WOMAC item responses recorded at M1 span a wider range than those recorded at follow-up, and it cannot be determined from the records whether the response format was identical across administrations. We searched the available documentation and found item-level responses only for the two follow-up administrations, so the M1 format cannot be established; equal numbers need not carry equal meaning if the response categories or their anchors differed. We therefore do not test or interpret longitudinal WOMAC change anywhere in this manuscript, and a reader wishing to compare WOMAC scores across assessments should treat such a comparison as uninterpretable rather than merely imprecise. This constraint does not affect the within-time-point analyses, in which both measures come from the same administration, and those analyses carry the study’s principal findings; but it does mean the study contributes nothing about how self-reported function changed over the observation period.5 and a maximum of 13 at M2, with 3 of 14 participants at 1 or below. A PROM operating over so narrow a range has little room to discriminate between participants, which limits what any association or agreement statistic computed against it can reveal, and it means the comparison is between a compressed self-report scale and objective measures that retained their full spread. A further constraint is the absence of any reference group. The study includes no non-arthroplasty comparison group and no age-matched healthy sample, so absolute mobility cannot be referenced to what would be expected of peers of the same age, and the analysis can describe only relative position within this cohort. Comorbidity, rehabilitation exposure, physical activity, muscle strength, contralateral knee status and analgesic use were not recorded and could not be examined as confounders. Agreement was assessed in standardised units and against a cut-off that is not externally validated. Finally, no correction for multiplicity can compensate for the number of outcomes examined relative to the number of participants, and all findings require independent confirmation.

Strengths. The study combines a patient-reported measure, standard functional tests and IMU-derived segmented variables in the same older cohort, reports agreement as well as association, and quantifies the stability of every principal estimate through confidence intervals, influence diagnostics and sensitivity analyses rather than relying on point estimates alone.

## 5. Conclusions

In adults aged 70 years or older assessed at least one year after unilateral TKA, WOMAC-based self-reported function was strongly associated with instrumented walking speed, cadence and 2MWT distance at the 2024 follow-up but not at first assessment, and individual participants frequently fell outside a standardised concordance band whichever validated objective outcome was used. Among the instrumented outcomes, segmented turning variables were the only ones to change detectably between the first two assessments. These are preliminary observations from a small single-centre cohort with heterogeneous postoperative timing, severe attrition and complete confounding between implant type and surgical approach. The prevalence of individual discordance was not stably estimable and its direction was not consistently asymmetric, so no prevalence figure or classification threshold from this study should be carried into practice. The findings support continued measurement of objective mobility alongside patient-reported outcomes after TKA in older recipients, and require independent confirmation in larger prospective cohorts before informing clinical interpretation.

## Figures and Tables

**Table 1:** Participant retention and availability of central outcomes at each assessment time point.

Time Point	Participants Assessed	TUG	2MWT	Walk Test	WOMAC Total	Interpretation
M1	25/25	22/25	22/25	22/25	25/25	Initial analytic cohort; M1 occurred 12–34 postoperative months
M2	14/25	14/25	14/25	14/25	14/25	2024 follow-up; approximately 18–24 months after M1 (dates not recorded)
M3	2/25	2/25	2/25	2/25	0/25	2025 follow-up; WOMAC not scorable (complete-case); attrition, not within-visit missing data

Note: The denominator of 25 is the initial analytic cohort. The effective denominator for each analysis is the number of participants with the relevant outcome at the relevant time point. WOMAC availability at M3 is 0/25 because neither returnee completed enough items for complete-case scoring. Exact assessment dates were not recorded, so inter-visit intervals are approximate.

**Table 2:** Continuous participant characteristics.

Variable	n	Mean ± SD	Median [IQR]	Minimum–Maximum
Age (years)	25	75.56 ± 4.00	74.00 [73.00–79.00]	70.00–84.00
Postoperative months at M1	25	15.84 ± 6.74	12.00 [12.00–18.00]	12.00–34.00
Weight (kg)	25	74.04 ± 10.85	70.00 [68.00–81.00]	55.00–98.00
Height (m)	25	1.58 ± 0.11	1.59 [1.50–1.68]	1.40–1.78
BMI (kg/m^2^)	25	29.80 ± 5.09	29.13 [24.77–32.88]	23.85–41.32

**Table 3:** Categorical participant characteristics.

Variable	Category	n	%
Sex	Female	16	64.0
Sex	Male	9	36.0
Prosthesis type	Fixed-bearing	15	60.0
Prosthesis type	Rotating-platform	10	40.0
Laterality	Right	14	56.0
Laterality	Left	11	44.0
Surgical approach	Transquadriceps	15	60.0
Surgical approach	Subvastus	10	40.0

**Table 4:** Distribution by age group and BMI category.

Variable	Category	n	%
Age group	70–74 years	13	52.0
Age group	75–79 years	7	28.0
Age group	≥80 years	5	20.0
BMI category	<25 kg/m^2^	8	32.0
BMI category	25.0–29.9 kg/m^2^	5	20.0
BMI category	30.0–34.9 kg/m^2^	9	36.0
BMI category	35.0–39.9 kg/m^2^	2	8.0
BMI category	≥40 kg/m^2^	1	4.0

**Table 5:** Comparison of M1 characteristics between participants reassessed at M2 and those not reassessed.

M1 Variable	n Reassessed	Reassessed at M2	n Not Reassessed	Not Reassessed	*p*	FDR *p*
Age (y)	14	74.86 ± 3.68	11	76.45 ± 4.39	0.336	0.736
BMI (kg/m^2^)	14	30.14 ± 4.05	11	29.36 ± 6.37	0.396	0.736
Weight (kg)	14	72.93 ± 8.79	11	75.45 ± 13.35	0.602	0.875
Height (m)	14	1.56 ± 0.12	11	1.61 ± 0.09	0.217	0.736
Months post-op at M1	14	17.21 ± 7.61	11	14.09 ± 5.26	0.186	0.736
Total TUG (s)	14	14.55 ± 3.19	8	17.20 ± 6.76	0.473	0.758
2MWT distance (m)	14	76.04 ± 20.61	8	73.29 ± 28.39	0.733	0.902
Walking speed (m/s)	14	0.94 ± 0.18	8	0.93 ± 0.17	0.733	0.902
Cadence (steps/min)	14	106.05 ± 15.64	8	100.50 ± 13.92	0.357	0.736
Mid-turn peak ang vel	14	100.83 ± 30.53	8	98.50 ± 27.20	0.868	0.972
WOMAC pain	14	2.57 ± 2.93	11	2.18 ± 2.64	0.911	0.972
WOMAC function	14	8.14 ± 8.06	11	14.27 ± 15.38	0.238	0.736
WOMAC total	14	10.71 ± 10.48	11	16.55 ± 17.74	0.351	0.736
Female sex	14	64.3%	11	63.6%	1.000	1.000
Rotating platform	14	50.0%	11	27.3%	0.414	0.736
Right side	14	71.4%	11	36.4%	0.116	0.736

Values are mean ± SD for continuous variables and percentages for binary variables. *p* values are from Mann–Whitney U tests or Fisher’s exact tests; FDR *p* values are Benjamini–Hochberg adjusted across the 16 comparisons. Non-significance here reflects low power and does not establish comparability.

**Table 6:** Functional and self-reported outcomes by assessment time point. The WOMAC columns are within-visit descriptives and are not to be read as change across time points (Section 2.5).

Variable	M1	M2	M3
Total TUG (s)	n = 22; 15.52 ± 4.82	n = 14; 16.30 ± 5.18	n = 2; 22.04 ± 7.01
TUG mid-turn duration (s)	n = 22; 4.22 ± 1.91	n = 14; 2.85 ± 1.01	n = 2; 3.71 ± 0.26
TUG mid-turn peak angular velocity (°/s)	n = 22; 99.98 ± 28.72	n = 14; 117.09 ± 34.62	n = 2; 74.70 ± 9.62
TUG mid-turn mean angular velocity (°/s)	n = 22; 50.52 ± 14.73	n = 14; 63.79 ± 21.57	n = 2; 41.45 ± 12.80
2MWT distance (m)	n = 22; 75.04 ± 23.10	n = 14; 84.40 ± 25.05	n = 2; 59.50 ± 21.64
2MWT average speed (m/s) ^a^	n = 22; 0.63 ± 0.19	n = 14; 0.70 ± 0.21	n = 2; 0.50 ± 0.18
2MWT stride-derived mean speed (m/s) ^b^	n = 22; 0.88 ± 0.31	n = 14; 0.86 ± 0.29	n = 2; 0.59 ± 0.23
Walking speed, walk test (m/s)	n = 22; 0.94 ± 0.17	n = 14; 0.88 ± 0.24	n = 2; 0.91 ± 0.14
Cadence, walk test (steps/min)	n = 22; 104.03 ± 14.95	n = 14; 98.64 ± 16.41	n = 2; 99.41 ± 24.43
WOMAC pain	n = 25; 2.40 ± 2.75	n = 14; 1.43 ± 1.45	n = 1; 5.00
WOMAC stiffness	n = 25; 0.04 ± 0.20	n = 14; 0.00 ± 0.00	n = 1; 0.00
WOMAC physical function	n = 25; 10.84 ± 11.97	n = 14; 4.64 ± 3.23	n = 0; not available
WOMAC total	n = 25; 13.28 ± 14.12	n = 14; 6.07 ± 4.46	n = 0; not available

Values are mean ± SD. ^a^ Average speed = 2MWT distance/nominal 120 s; the realised test duration had a median of 118.5 s (range 110–124 s) at M1 and was not recorded at later visits. ^b^ Stride-derived mean speed is computed by the device from stride length and cadence and is not interchangeable with average speed or with walk-test speed. WOMAC scores are complete-case; nominal scale ranges are not stated because the response format may have differed between administrations (Section 2.5).

**Table 7:** Paired M1–M2 comparison of the instrumented outcomes using the Wilcoxon signed-rank test, with bootstrap confidence intervals.

Variable	n	Median Δ	95% CI for Median Δ	*p*	FDR *p*	dz (95% CI)
Total TUG time (s)	14	0.07	−1.05 to 3.78	0.268	0.306	0.42 (−0.08 to 0.89)
TUG mid-turn duration (s)	14	−0.80	−0.93 to −0.28	0.011	0.036	−0.64 (−1.95 to −0.13)
TUG mid-turn peak angular velocity (°/s)	14	16.55	8.00 to 30.10	0.013	0.036	0.79 (0.25 to 1.83)
TUG mid-turn mean angular velocity (°/s)	14	7.40	1.30 to 14.15	0.009	0.036	0.64 (0.21 to 1.27)
2MWT distance (m)	14	12.80	6.10 to 19.60	0.091	0.145	0.46 (−0.07 to 2.27)
2MWT stride-derived mean speed (m/s)	14	0.01	−0.08 to 0.09	0.975	0.975	−0.17 (−0.66 to 0.50)
Walking speed (m/s)	14	−0.04	−0.14 to 0.02	0.142	0.189	−0.34 (−1.01 to 0.16)
Cadence (steps/min)	14	−6.77	−12.97 to −1.54	0.058	0.116	−0.59 (−1.30 to −0.11)

FDR, false discovery rate; dz, paired standardised effect size. Confidence intervals are 2.5th–97.5th percentile bootstrap intervals from 10,000 resamples. Negative duration changes indicate faster performance. The FDR family comprised the eight instrumented outcomes listed. Longitudinal WOMAC change is not tested and does not appear in this table; see Section 2.5.

**Table 8:** Observed M1–M2 changes benchmarked against measurement error and clinical-relevance thresholds.

Outcome	Observed Change (Median Δ)	Threshold	Basis of Threshold	Interpretation
Total TUG (s)	+0.07 (mean +1.75)	2.27	MDC_95_ in TKA [26]	Within measurement error
TUG mid-turn duration (s)	−0.80	0.96	0.5 × SD (M1) [39]	Below threshold
TUG mid-turn peak angular velocity (°/s)	+16.55	14.36	0.5 × SD (M1) [39]	Exceeds threshold by 2.19
TUG mid-turn mean angular velocity (°/s)	+7.40	7.37	0.5 × SD (M1) [39]	Exceeds threshold by 0.03
2MWT distance (m)	+12.80	14.96	MDC_95_ in TKA [26]	Below MDC_95_
Walking speed (m/s)	−0.04	0.05/0.10	Small/substantial meaningful change [40]	Below the smallest meaningful change
Cadence (steps/min)	−6.77	7.48	0.5 × SD (M1) [39]	Below threshold

MDC_95_, minimal detectable change at the 95% confidence level; MID, minimal important difference. Distribution-based thresholds were computed as half of the M1 standard deviation of the available-case cohort (Table 6). The 2MWT average speed is not benchmarked separately because it is an exact linear transformation of distance and must be judged against the MDC of the 2MWT rather than against gait-speed anchors, which were derived for steady-state walking over short distances. Margins are reported for the two turning variables that exceed their thresholds; for mid-turn mean angular velocity the margin is 0.03°/s and the confidence interval for the median change (1.30 to 14.15°/s) does not resolve whether the threshold is exceeded. Benchmarks are confined to the instrumented outcomes; WOMAC change is not benchmarked, because the comparison would presuppose a common metric across administrations that cannot be established (Section 2.5).

**Table 9:** Spearman correlations between instrumented mobility and the total WOMAC score, with confidence intervals and influence diagnostics.

Time Point	Objective Outcome	n	rho	95% Bootstrap CI	*p*	FDR *p*	Leave-One-Out Range	Significant in All Refits
M1	Total TUG time	22	+0.297	−0.128 to 0.640	0.179	0.492	+0.216 to +0.392	No
M1	2MWT distance	22	−0.138	−0.555 to 0.323	0.540	0.540	−0.211 to −0.026	No
M1	2MWT stride-derived mean speed	22	−0.216	−0.624 to 0.254	0.335	0.492	−0.300 to −0.116	No
M1	Walking speed, walk test	22	−0.191	−0.596 to 0.286	0.394	0.492	−0.303 to −0.079	No
M1	Cadence, walk test	22	−0.251	−0.645 to 0.213	0.259	0.492	−0.386 to −0.157	No
M2	Total TUG time	14	+0.569	−0.013 to 0.896	0.034	0.042	+0.461 to +0.711	No
M2	2MWT distance	14	−0.644	−0.901 to −0.124	0.013	0.022	−0.747 to −0.559	Yes
M2	2MWT stride-derived mean speed	14	−0.531	−0.901 to 0.103	0.051	0.051	−0.702 to −0.422	No
M2	Walking speed, walk test	14	−0.762	−0.973 to −0.307	0.002	0.004	−0.915 to −0.710	Yes
M2	Cadence, walk test	14	−0.761	−0.949 to −0.345	0.002	0.004	−0.860 to −0.701	Yes

Confidence intervals are 2.5th–97.5th percentile bootstrap intervals from 10,000 resamples; Fisher z-transformed intervals are reported in Table S3. The leave-one-out range is the span of the coefficient when each participant is removed in turn. FDR adjustment is within time point. Because Spearman’s coefficient is invariant under monotone transformation, the coefficient for 2MWT average speed is identical to that for 2MWT distance and is not listed separately.

**Table 10:** Individual-level agreement between each validated objective outcome and the WOMAC total score.

Time Point	Objective Outcome	n	Concordant	Objective Better	Self-Report Better	% Outside Band	% Outside Across Cut-Offs
M1	Total TUG time (s)	22	8	7	7	63.6	13.6–95.5
M1	2MWT distance (m)	22	8	7	7	63.6	22.7–86.4
M1	Walking speed (m/s)	22	6	8	8	72.7	13.6–100.0
M2	Total TUG time (s)	14	8	2	4	42.9	7.1–85.7
M2	2MWT distance (m)	14	6	3	5	57.1	7.1–78.6
M2	Walking speed (m/s)	14	8	2	4	42.9	7.1–71.4

Classification uses the difference between the standardised objective outcome and the standardised inverted WOMAC total score within each time point Equation (1). The concordance band is ±0.5 standardised units. The final column gives the range of the discordant proportion across cut-offs from 0.1 to 1.5 standardised units; no cut-off is externally validated for this comparison. Proportions at ±0.3 and ±1.0 standardised units are given in Section 3.8, and the classification anchored on the physical-function subscale in Supplementary Table S4.

## Data Availability

The de-identified data supporting the reported results are available from the corresponding author on reasonable request, subject to institutional ethics approval and privacy restrictions.

## Supplementary Materials

The following supporting information can be downloaded at: https://www.mdpi.com/article/10.3390/jfmk11040420/s1, Table S1: individual values at each assessment time point for the two participants reassessed at M3; Table S2: exploratory comparison between fixed-bearing and rotating-platform implants; Table S3: Spearman correlations with both bootstrap and Fisher z-transformed confidence intervals; Table S4: correlations with the WOMAC physical-function subscale and individual-level agreement anchored on that subscale; Figure S1: individual trajectories of total TUG time for the two M3 returnees.

## Abbreviations

The following abbreviations are used in this manuscript:

2MWT	2-Minute Walk Test
BMI	Body mass index
FB	Fixed-bearing
FDR	False discovery rate
IMU	Inertial measurement unit
IQR	Interquartile range
M1	First available assessment
M2	2024 follow-up; approximately 18–24 months after M1
M3	2025 follow-up
MDC_95_	Minimal detectable change at the 95% confidence level
MID	Minimal important difference
PROM	Patient-reported outcome measure
RP	Rotating-platform
SD	Standard deviation
SEM	Standard error of measurement
TKA	Total knee arthroplasty
TUG	Timed Up and Go
WOMAC	Western Ontario and McMaster Universities Osteoarthritis Index

## Appendix A

### Appendix A.1. Core Variables Prioritised for Analysis

All original variables were retained in the analytic database. For the manuscript, priority was given to outcomes that are clinically interpretable and comparable across time points: total TUG time and the segmented mid-turn variables; 2MWT distance and the two derived speed variables; walking speed and cadence from the instrumented walk test; and the WOMAC domains. Acceleration fields with evident input artefacts were not used as confirmatory outcomes.

### Appendix A.2. Computation of the Individual-Level Agreement Difference

For each assessment time point j and participant i, the objective outcome and the inverted WOMAC total score were standardised using the mean and standard deviation of the participants assessed at that time point who had both measures available, giving z-scores oriented so that higher values indicate better status. The agreement difference is the objective z-score minus the inverted-WOMAC z-score, as given in Equation (1). Because both terms are standardised within time point, the mean difference is zero by construction at every visit, and the classification therefore describes the dispersion of individual differences rather than any systematic offset. No composite of multiple objective outcomes was formed: each of the three validated outcomes enters the computation separately, so no weighting decision arises, and the three resulting classifications are reported side by side as a check on whether the finding depends on the choice of outcome.
