# Supplementary material for: Dissociation Between Instrumented Mobility and Self-Reported Function in Adults Aged 70 Years or Older After Unilateral Total Knee Arthroplasty: A Preliminary Longitudinal Study Using an Inertial Sensor and the WOMAC
Source: J Funct Morphol Kinesiol. 2026 Oct 14;11(4):420. doi: 10.3390/jfmk11040420 (PMC13692415; doi:10.3390/jfmk11040420)
Supplement: Supplementary file 1 [file jfmk-11-00420-s001.zip › jfmk-4521482-supplementary.pdf]

Dissociation between Instrumented Mobility and Self-Reported Function in Adults Aged 70 Years or Older after Unilateral Total Knee Arthroplasty: A Preliminary Longitudinal Study Using an Inertial Sensor and the WOMAC

All values were recomputed from the source records. Participants are identified by pseudonymous labels; no identifying information is included. Assessment time points are M1, the first available assessment; M2, the 2024 follow-up, approximately 18 to 24 months after M1; and M3, the 2025 follow-up. Exact assessment dates were not recorded and all intervals are approximate.

Table S1. Individual values at each assessment time point for the two participants reassessed at M3 (labelled A and B). With two participants these values are descriptive and support no inference. [a] WOMAC values are within-visit descriptives only. Differences between WOMAC columns are not interpretable as change, because the response format used at M1 cannot be established from the available records (main text, Section 2.5). WOMAC scores are complete-case: a domain is reported only where every item in it was answered, and returnee A answered 2 of the 24 items at M3 and returnee B 23 of 24, so no WOMAC total is available at M3 for either.

Variable	A: M1	A: M2	A: M3	B: M1	B: M2	B: M3
Total TUG (s)	12.84	12.83	17.08	17.81	17.92	27.00
TUG mid-turn duration (s)	2.95	2.67	3.53	3.65	2.39	3.90
TUG mid-turn peak angular velocity (°/s)	116.30	124.30	81.50	89.60	119.70	67.90
TUG mid-turn mean angular velocity (°/s)	60.70	63.50	50.50	52.10	64.10	32.40
2MWT distance (m)	77.50	90.90	74.80	56.00	67.80	44.20
2MWT stride-derived mean speed (m/s)	0.87	0.93	0.76	0.61	0.67	0.43
Walking speed, walk test (m/s)	1.01	0.92	1.01	0.77	0.79	0.81
Cadence, walk test (steps/min)	118.55	117.01	116.69	89.63	84.35	82.14
WOMAC pain [a]	1.00	0.00	not scorable	5.00	2.00	5.00
WOMAC stiffness [a]	0.00	0.00	not scorable	0.00	0.00	0.00
WOMAC physical function [a]	10.00	5.00	not scorable	12.00	6.00	not scorable
WOMAC total [a]	11.00	5.00	not scorable	17.00	8.00	not scorable

Table S2. Exploratory comparison between fixed-bearing (FB) and rotating-platform (RP) implants, within time point. Implant type is completely confounded with surgical approach in this cohort (all FB procedures transquadriceps, all RP subvastus), so these comparisons contrast two surgical packages rather than two bearing designs. p values are from Mann-Whitney U tests and none was significant after false-discovery-rate adjustment. The absence of a detected difference is not evidence of equivalence: prosthesis type was not a prespecified objective and each subgroup contains 7 participants at M2.

Time point	Outcome	n FB	Median FB	n RP	Median RP	p	Cliff's delta
M1	Total TUG (s)	13	14.64	9	15.14	0.333	-0.26
M1	2MWT distance (m)	13	76.30	9	70.20	0.423	+0.21
M1	Walking speed (m/s)	13	0.94	9	0.95	0.947	+0.03
M1	WOMAC total	15	9.00	10	5.50	0.359	+0.23
M2	Total TUG (s)	7	15.75	7	14.09	0.902	+0.06
M2	2MWT distance (m)	7	90.00	7	82.60	0.710	+0.14
M2	Walking speed (m/s)	7	0.79	7	0.91	0.306	-0.35
M2	WOMAC total	7	8.00	7	5.00	0.898	+0.06

Table S3. Spearman correlations between instrumented mobility and the total WOMAC score, computed within time point, with both bootstrap and Fisher z-transformed confidence intervals. Bootstrap intervals are 2.5th to 97.5th percentile intervals from 10,000 resamples and are the intervals quoted in the main text.

Time point	Objective outcome	n	rho	95% bootstrap CI	95% Fisher z CI
M1	Total TUG time	22	+0.297	-0.128 to 0.640	-0.142 to 0.639
M1	2MWT distance	22	-0.138	-0.555 to 0.323	-0.529 to 0.301
M1	2MWT stride-derived mean speed	22	-0.216	-0.624 to 0.254	-0.584 to 0.226
M1	Walking speed, walk test	22	-0.191	-0.596 to 0.286	-0.567 to 0.251
M1	Cadence, walk	22	-0.251	-0.645 to 0.213	-0.608 to 0.191

	test				
M2	Total TUG time	14	+0.569	-0.013 to 0.896	0.055 to 0.845
M2	2MWT distance	14	-0.644	-0.901 to -0.124	-0.875 to -0.172
M2	2MWT stride-derived mean speed	14	-0.531	-0.901 to 0.103	-0.828 to -0.001
M2	Walking speed, walk test	14	-0.762	-0.973 to -0.307	-0.920 to -0.388
M2	Cadence, walk test	14	-0.761	-0.949 to -0.345	-0.920 to -0.386

Table S4. Analyses anchored on the WOMAC physical-function subscale instead of the total score. Panel A gives within-time-point Spearman correlations between instrumented mobility and the subscale, with bootstrap confidence intervals, FDR-adjusted p values and leave-one-out influence diagnostics. Panel B gives individual-level agreement using the same ±0.5 standardised concordance band applied to the subscale. Correlations are computed within time point and are not comparisons across time points.

**A. Within-time-point correlations with the WOMAC physical-function subscale**								
Time point	Objective outcome	n	rho	95% bootstrap CI	p	FDR p	Leave-one-out range	Significant in all refits
M1	Total TUG time	22	+0.270	-0.128 to 0.588	0.225	0.555	+0.186 to +0.349	No
M1	2MWT distance	22	-0.133	-0.532 to 0.319	0.555	0.555	-0.197 to -0.021	No
M1	Walking speed, walk test	22	-0.161	-0.547 to 0.272	0.473	0.555	-0.259 to -0.044	No
M1	Cadence, walk test	22	-0.157	-0.564 to 0.267	0.485	0.555	-0.272 to -0.048	No
M2	Total TUG time	14	+0.532	-0.060 to 0.857	0.050	0.050	+0.418 to +0.643	No
M2	2MWT distance	14	-0.600	-0.899 to -0.025	0.023	0.031	-0.709 to -0.510	No
M2	Walking speed, walk test	14	-0.782	-0.961 to -0.342	0.001	0.004	-0.914 to -0.728	Yes
M2	Cadence, walk test	14	-0.724	-0.914 to -0.307	0.003	0.007	-0.801 to -0.665	Yes
**B. Individual-level agreement anchored on the WOMAC physical-function subscale**								
Time point	Objective outcome	n	Concordant	Objective better	Self-report	% outside		

					better	band	
M1	Total TUG time (s)	22	6	8	8	72.7	
M1	2MWT distance (m)	22	8	7	7	63.6	
M1	Walking speed (m/s)	22	5	8	9	77.3	
M2	Total TUG time (s)	14	6	3	5	57.1	
M2	2MWT distance (m)	14	6	3	5	57.1	
M2	Walking speed (m/s)	14	8	3	3	42.9	

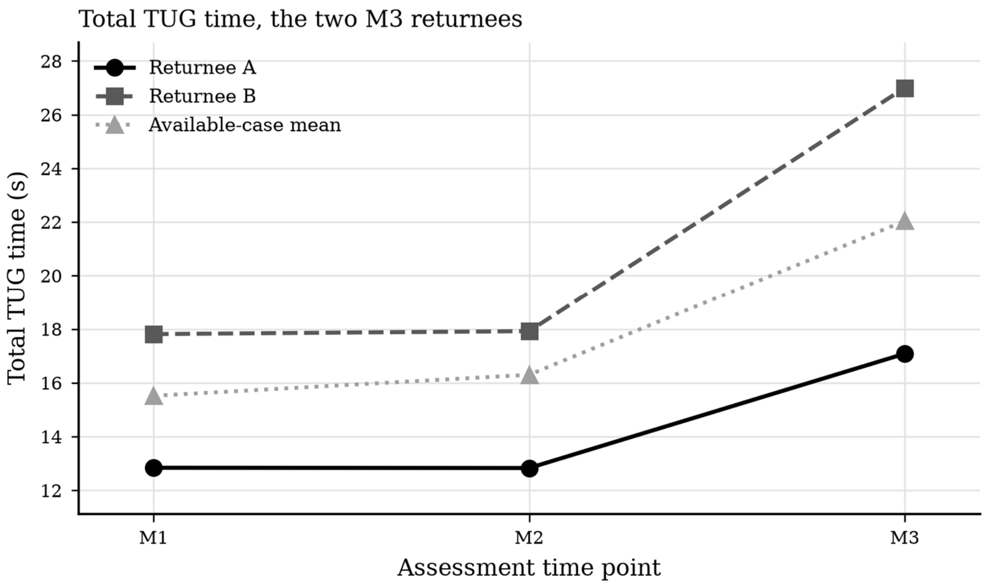

Figure S1. Individual trajectories of total TUG time for the two participants reassessed at M3, with the available-case mean at each time point. The two returnees are labelled A and B; the available-case mean is computed on 22, 14 and 2 participants respectively and the three points are therefore not a common trajectory.
